# Comprehensive Analysis of Transcriptomics and Genetic Alterations Identifies Potential Mechanisms Underlying Anthracycline Therapy Resistance in Breast Cancer

**DOI:** 10.3390/biom12121834

**Published:** 2022-12-08

**Authors:** Zihao Liu, Jingbo Gao, Ran Gu, Yu Shi, Hong Hu, Jianlan Liu, Jiefeng Huang, Caineng Zhong, Wenbin Zhou, Yaping Yang, Chang Gong

**Affiliations:** 1 Breast Tumor Center, Sun Yat-sen Memorial Hospital, Sun Yat-sen University, Guangzhou 510120, China; 2 Department of Breast and Thyroid Surgery, The Second Clinical Medical College of Jinan University, The First Affiliated Hospital of Southern University of Science and Technology, Shenzhen People’s Hospital, Shenzhen 518020, China; 3 Department of Pathology, The Second Clinical Medical College of Jinan University, The First Affiliated Hospital of Southern University of Science and Technology, Shenzhen People’s Hospital, Shenzhen 518020, China

**Keywords:** anthracycline, resistance, breast cancer

## Abstract

Anthracycline is a mainstay of treatment for breast cancer patients because of its antitumor activity. However, anthracycline resistance is a critical barrier in treating breast cancer. Thus, it is of great importance to uncover the molecular mechanisms underlying anthracycline resistance in breast cancer. Herein, we integrated transcriptome data, genetic alterations data, and clinical data of The Cancer Genome Atlas (TCGA) to identify the molecular mechanisms involved in anthracycline resistance in breast cancer. Two hundred and four upregulated genes and 1376 downregulated genes were characterized between the anthracycline-sensitive and anthracycline-resistant groups. It was found that drug resistance-associated genes such as ABCB5, CYP1A1, and CYP4Z1 were significantly upregulated in the anthracycline-resistant group. The gene set enrichment analysis (GSEA) suggested that the P53 signaling pathway, DNA replication, cysteine, and methionine metabolism pathways were associated with anthracycline sensitivity. Somatic *TP53* mutation was a common genetic abnormality observed in the anthracycline-sensitive group, while *CDH1* mutation was presented in the anthracycline-resistant group. Immune infiltration patterns were extremely different between the anthracycline-sensitive and anthracycline-resistant groups. Immune-associated chemokines and cytokines, immune regulators, and human leukocyte antigen genes were significantly upregulated in the anthracycline-sensitive group. These results reveal potential molecular mechanisms associated with anthracycline resistance.

## 1. Introduction

Breast cancer has become the most commonly diagnosed cancer among females worldwide and the incidence of breast cancer is still increasing, with nearly 2 million patients requiring chemotherapy by 2040. The tremendous development of systemic chemotherapy consequently improves the clinical outcomes of patients with breast cancer. The systemic chemotherapeutic agents used for breast cancer include anthracyclines, cyclophosphamide, taxanes, fluorouracil, and others. Among these agents, anthracyclines display robust antitumor activity in breast cancer. However, several patients still suffer from anthracycline resistance, leading to poor survival. It is reported that over 90% of treatment failure in patients with metastatic breast cancer are associated with anthracycline resistance [1,2].Consequently, because of therapy resistance, breast cancer patients are usually confronted with disease progression even if they receive intensive treatment.

Anthracyclines include doxorubicin (also known as adriamycin), epirubicin, doxorubicinum, and liposomal forms of these agents. Anthracycline was firstly isolated from *Streptomyces peucetius*. For mammalian cells, topoisomerase (Top) IIα enzyme known as a DNA replication regulator is specifically expressed by proliferation cells. Anthracycline can bind to Top IIα and disrupt DNA replication by forming an anthracycline-Top IIα-DNA complex. Anthracycline can also disrupt the normal nucleosome structure as well as histone-DNA association [3,4,5]. Besides, anthracycline can bind with mitochondrial membrane lipid, inhibit normal function of electron transport chain, and lead to reactive oxygen species (ROS) generation. Anthracycline also functions as an electron acceptor and iron chelator that influences ROS metabolism [3,4,5]. Overall, anthracycline treatment causes irreversible DNA damage, oxidative stress, and apoptotic cell death. Despite the robust antitumor activity of anthracyclines, breast cancer cells may still develop anthracycline resistance. To date, many factors have been reported to be involved in anthracycline resistance. It is reported that the alteration of Top II protein has an impact on the binding affinity between anthracycline and Top II. The increased expression of ATP-binding cassette subfamily B member 1 (ABCB1) acts as an efflux pump extrudes anthracycline [6,7,8]. Other drug metabolism machineries such as lysosomal clearance, glutathione S-transferase, and drug transporter have been proposed to regulate anthracycline resistance [9,10]. In addition, genetic mutations were also proved to promote anthracycline resistance [11]. Since anthracycline resistance is one of the main reasons limiting the successful treatment of breast cancer, understanding the mechanisms leading to anthracycline resistance can improve treatment strategies for breast cancer and improve the survival of patients.

Up to now, few studies performed comprehensive analyses to elucidate the potential mechanisms of anthracycline resistance in breast cancer. In this study, we performed a comprehensive analysis on the transcriptome data, genetic alterations, and immune infiltration status in anthracycline-resistant samples of breast cancer. Our study increases the understanding of anthracycline resistance and provides potential diagnostic and prognostic markers for breast cancer patients with anthracycline resistance.

## 2. Materials and Methods

### 2.1. Data Download and Analysis

Gene counts and the relative expression matrix were originated from The Cancer Genome Atlas (TCGA) database. Eighty-six breast cancer patients who had complete responses to anthracycline treatment and seven patients who had resistance to anthracycline treatment were collected. The corresponding clinical information including breast cancer subtypes and survival outcomes was also obtained. If there were no clinically significant changes after 2–3 cycles of chemotherapy, we should suspect the emergence of chemoresistance. Chemoresistance was defined as clinical progressive disease at the first restaging of breast cancer or clinical stable disease for less than 6 months [12,13].

### 2.2. Differentially Expressed Genes Analysis

The gene count matrix, which was downloaded from TCGA, was used to calculate the differentially expressed genes through the DESeq2 R package. The significantly upregulated genes were those with log_2_FC > 1.0 and *p* < 0.05, while the significantly downregulated genes were those with log_2_FC < −1.0 and *p* < 0.05.

### 2.3. Genetic Alterations and Tumor Mutation Burden (TMB) Estimation

The masked somatic mutation data were obtained, analyzed, and visualized using the maftools in the R package. The TMB was calculated by the somatic mutation per megabase of the genomic sequence. The low-TMB and high-TMB groups were classified according to the median TMB. The TMB data were merged with the corresponding survival information via the TCGA ID number.

### 2.4. Immune Infiltration Analysis

The relative expression matrix, which was downloaded from the TCGA database, was used to calculate the absolute infiltration score of tumor samples by CIBERSORT, XCELL, and MCP-counter. Each immune cell fraction was merged through the corresponding TCGA ID number.

### 2.5. Gene Set Enrichment Analysis (GSEA)

The GSEA was performed using GSEA software. The hallmarks gene sets and Kyoto Encyclopedia of Genes and Genomes (KEGG) pathway gene sets were downloaded from the GSEA official website (https://www.gsea-msigdb.org/gsea/, accessed on 30 August 2022). The relative expression matrix was used for GSEA.

### 2.6. Statistical Analysis

All the statistical analyses were performed using the GraphPad Prism 5 software. The Kaplan-Meier method with a log-rank test was used to compare the prognosis of breast cancer patients. A two-tailed Student’s *t*-test or one-way analysis of variance (ANOVA) with a Tukey’s post hoc test were used for the intergroup comparison of normally distributed continuous variables. A Wilcoxon signed-rank test was used for immune cell fraction comparison. In all the analyses, *p* < 0.05 was considered statistically significant.

## 3. Results

### 3.1. Anthracycline-Sensitive (Anthracycline-S) and -Resistant (Anthracycline-R) Tumors Display Distinct Transcriptomic Profiles

**Figure 1:**
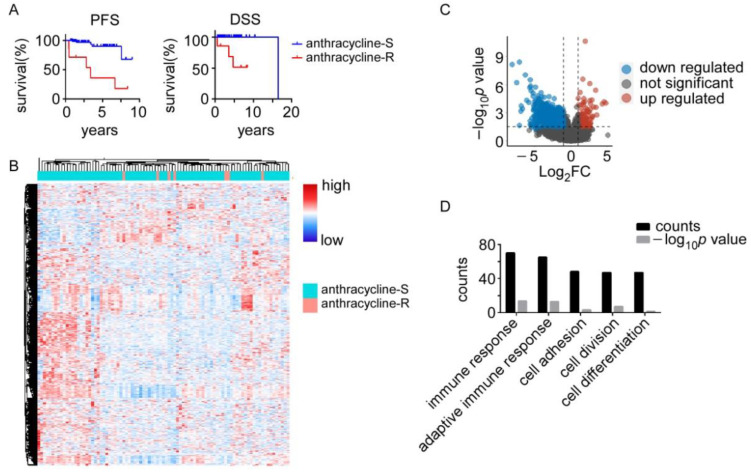
Thetranscriptomic difference between the anthracycline-sensitive (anthracycline-S) and anthracycline-resistant (anthracycline-R) patients. (**A**). Progression-free survival (PFS) and disease-specific overall survival (DSS) between the anthracycline-S (*n* = 86) and anthracycline-R (*n* = 7) patients. (**B**). Heatmap of differentially expressed genes between the anthracycline-S and anthracycline-R patients. (**C**). Volcano plot of differentially expressed genes between the anthracycline-S and anthracycline-R patients. (**D**). Gene ontology analysis of differentially expressed genes between the anthracycline-S and anthracycline-R patients.

The chemotherapy regimens and patients’ clinical response data were retrieved from the TCGA database. The most commonly used systemic chemotherapeutic agents for breast cancer treatment are summarized in Supplementary Table S1. The patients treated with anthracyclines including adriamycin, doxorubicin, doxorubicinum, and epirubicin were collected. The anthracycline-R patients exhibited short progression-free survival and poor disease-specific overall survival than the patients who had clinical complete response to anthracycline (Figure 1A). We then compared transcriptomic profiles between the anthracycline-S and anthracycline-R patients and found 204 upregulated genes (log_2_FC > 1, *p* < 0.05) and 1376 downregulated genes (log_2_FC < −1, *p* < 0.05; Figure 1B,C and Supplementary Table S2). The gene ontology analysis showed that these genes with significantly different expression were involved in the immune response, adaptive immune response, cell adhesion, cell division, and cell differentiation via the DAVID database(Figure 1D and Supplementary Table S3).

### 3.2. Signaling Pathways Involved in Anthracycline Sensitivity

**Figure 2:**
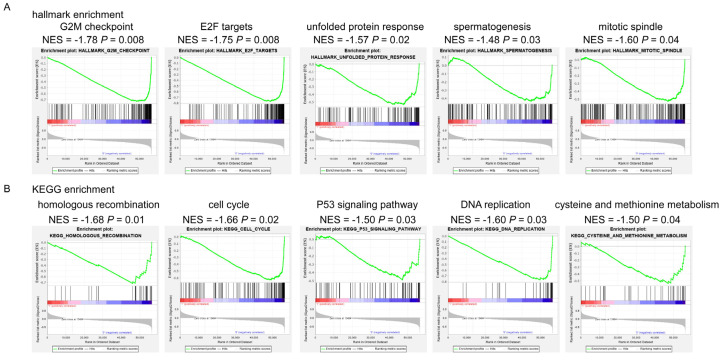
Gene set enrichment analysis (GSEA) of anthracycline-sensitive (anthracycline-S)and anthracycline-resistant (anthracycline-R) patients. (**A**). GSEA on hallmarkers in the anthracycline-S patients. G2M checkpoint, E2F targets, unfolded protein response, spermatogenesis, and mitotic spindle hallmarkers were enriched in the anthracycline-S samples. (**B**). GSEA on Kyoto Encyclopedia of Genes and Genomes (KEGG) pathways in the anthracycline-S patients. Homologous recombination, cell cycle, P53 signaling pathway, DNA replication, and cycteine and methionine metabolism pathways were enriched in the anthracycline-S patients.

For further gene ontology analysis, we performed GSEA onhallmarkers and KEGG signalingpathways. For hallmarkers, we found that the anthracycline-S patientshad enrichments of G2M checkpoint, E2F targets, unfolded protein response, spermatogenesis, mitotic spindle, and other hallmarkers (Figure 2A and Supplementary Table S4). For KEGG signaling pathways, we found that homologous recombination, cell cycle, P53 signaling pathway, DNA replication, cysteine and methionine metabolism, and other KEGG pathways were significantly enriched in the anthracycline-S patients (Figure 2B, Supplementary Table S4). In contrast, the anthracycline-R patientshad no significant enrichments of hallmarkers or KEGG pathways (Supplementary Table S4).

### 3.3. Somatic Genetic Alterations in the Anthracycline-S and Anthracycline-R Tumors

**Figure 3:**
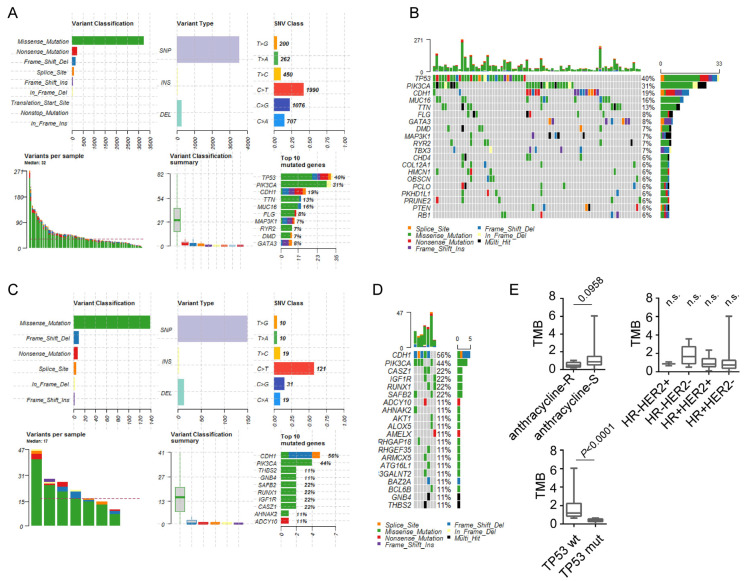
Genetic alterations of the anthracycline-sensitive (anthracycline-S) and anthracycline-resistant (anthracycline-R) patients. (**A**). Summary of mutations in the anthracycline-S patients. (**B**). Waterfall plot of top 20 mutation genes in the anthracycline-S patients. (**C**). Summary of mutations in the anthracycline-R patients. (**D**). Waterfall plot of top 20 mutation genes in the anthracycline-R patients. (**E**). Tumor mutation burden (TMB) in the anthracycline-S and anthracycline-R patients, in different subtypes of breast cancer and in the *TP53* mutated (*TP53* mut) and wild-type (*TP53* wt) patients.

The somatic mutation data of the 86 anthracycline-S patients and 7 anthracycline-R patients were analyzed. Among these mutations, missense mutations were the most common mutations in the two groups (Figure 3A–D). The most common missense mutation type was single nucleotide polymorphism (SNP) and C > T transversion was the most common type of single nucleotide variant (SNV) in the anthracycline-S and anthracycline-R patients (Figure 3A–D). For the anthracycline-S group, the top 10 mutated genes were *TP53*, *PIK3CA*, *CDH1*, *MUC16*, *TTN*, *FLG*, *GATA3*, *DMD*, and *MAP3K1* (Figure 3A,B). Consistently, the P53 signaling pathway was significantly enriched in the anthracycline-S group analyzed by GSEA on KEGG pathways. Interestingly, the previous reports indicated that the *TP53* mutation conferred anthracycline resistance to breast cancer patients and resulted in a poor prognosis [14,15,16,17]. As for the anthracycline-R group, the top 10 mutated genes were *CDH1*, *PIK3CA*, *THBS2*, *GNB4*, *SAFB2*, *RUNX1*, *IGF1R*, *AHNAK2*, and *ADCY10* (Figure 3C,D). Since TMB is a promising predictor for outcomes after immunotherapy and other therapies, we also analyzed TMB between the anthracycline-S and anthracycline-R groups. The TMB was marginally lower in the anthracycline-R group than in the anthracycline-S group, but not correlated with breast cancer subtypes (Figure 3E). In the anthracycline-S group, the TMB was significantly higher in the *TP53*wild-type group than in the *TP53* mutated group (*p* < 0.0001;Figure 3E).

### 3.4. Immune Infiltration Patterns in the Anthracycline-S and Anthracycline-R Tumors

**Figure 4:**
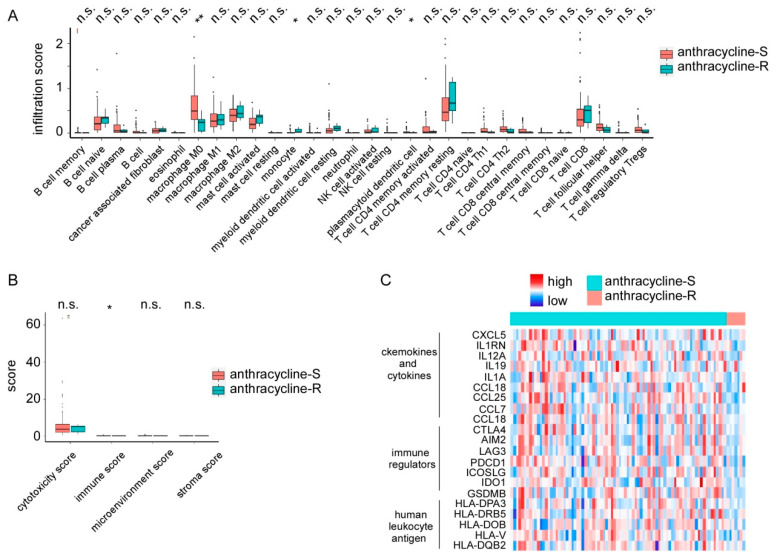
Immune status in anthracycline-S and anthracycline-R patients. (**A**). Immune infiltration estimated by CIBERSORT in anthracycline-S and anthracycline-R patients. (**B**). Cytotoxicity score, immune score, microenvironment score and stroma score estimated by XCELL and MCP-counter in anthracycline-S and anthracycline-R patients. (**C**). Differentially expressed immune associated genes in anthracycline-S and anthracycline-R patients. n.s. not significant, * *p* < 0.05, ** *p* < 0.01.

To further evaluate the populations of immune cells and the enrichment of immune signatures involved in anthracycline resistance, CIBERSORT and XCELL algorithms were performed. The anthracycline-S group showed a high level of M0 macrophage and plasmacytoid dendritic cells, while the anthracycline-R group showed a high level of monocytes (Figure 4A). Besides, the anthracycline-S group showed a high level of immune score (Figure 4B). For further immune score analysis, the immune-associated genes were extracted from differentially expressed genes (Figure 4C and Supplementary Table S2). The heatmap showed that chemokines and cytokines including CXCL5, IL12A, IL-6, IL19, CCL18, CCL25, and CCL7; immune regulators including CTLA4, AIM2, LAG3, PDCD1, ICOSLG, IDO1, and GSDMB; and human leukocyte antigens including HLA-DPA3, HLAD-DRB5, HLA-DOB, HLA-V, and HLA-DQB2 were significantly upregulated in the anthracycline-S group [18,19].

## 4. Discussion

Anthracyclines (such as adriamycin, doxorubicin, doxorubicinum, and epirubicin) are fundamental chemotherapy drugs for breast cancer treatment. Although anthracycline-based chemotherapy shows tremendous anti-tumor activity, some patients still suffer from anthracycline resistance. The failure of anthracycline-based chemotherapy may be associated with poor prognosis in breast cancer patients [1,2]. However, the molecular mechanisms underlying anthracycline resistance remains unclear. It is of great importance to reveal the potential mechanisms promoting anthracycline resistance.

In this study, we analyzed transcriptome data, genetic alterations, and the immune status of the anthracycline resistance samples to uncover the potential mechanisms involved in anthracycline resistance. Generally, 204 upregulated genes and 1376 downregulated genes were characterized and the gene ontology analysis of these genes showed that they were involved in immune response, cell adhesion, cell division, and cell differentiation. In addition, the GSEA analysis showed that the cell cycle processes such as the G2M checkpoint, E2F targets, cell cycle and mitotic spindle, and DNA regulation processes such as homologous recombination, P53 signaling pathway, and DNA replication were associated with anthracycline sensitivity. It is not surprising that cell cycle processes are associated with anthracycline sensitivity, as anthracycline inhibits DNA Top II activity, mediates cell cycle arrest, and induces oxidative stress, which in turn mediates cell death [3,20]. Interestingly, the anthracycline-S samples had the enrichment of P53 signaling pathway and nearly 40% of the anthracycline-S samples had *TP53* mutation while none of the anthracycline-R samples had *TP53* mutation. The previous reports showed that *TP53* mutation was associated with anthracycline sensitivity [14,15,16,17], while some indicated that *TP53* mutation conferred anthracycline resistance to breast cancer patients and resulted in a poor prognosis [21,22,23,24]. Our results emphasized the important role of *TP53* mutation in anthracycline sensitivity. As for anthracycline resistance, 56% of anthracycline-R patients were found to have *CDH1* mutation. The lifetime risk of breast cancer in the *CDH1* mutation carriers ranges from 40% to 50% [25]. The *CDH1* mutation and abnormal expression of CDH1 are associated with therapy resistance in several types of cancer [26,27].

Through CIBERSORT, XCELL, and MCP-counter, the immune status was compared between the anthracycline-S and anthracycline-R groups. The immune score as well as some immune-associated genes such as chemokines, cytokines, immune regulators, and human leukocyte antigens were significantly higher in the anthracycline-S group. Previous studies reported that anthracycline-based chemotherapy was known to induce cytotoxic T cell proliferation and enhance the anti-tumor immune response by turning a “cold” tumor into a “hot” one [3,28].Besides, several studies suggested that the combination of anthracycline and immune checkpoint inhibitors might improve survival [29,30,31]. Our results suggested that anti-tumor-associated immunological processes were much more active in the anthracycline-S group than in the anthracycline-R group and that the anthracycline-R samples exhibit restricted anti-tumor immunity from the very beginning of anthracycline treatment. Besides, our results suggested that the detection of markers such aschemokines and cytokines (CXCL5, IL12A, IL19, CCL18, CCL25, and CCL7),immune regulators (CTLA4, AIM2, LAG3, PDCD1, ICOSLG, IDO1, and GSDMB),and human leukocyte antigens(HLA-DPA3, HLAD-DRB5, HLA-DOB, HLA-V, and HLA-DQB2)might predict anthracycline resistance.

In our analysis, we used the TCGA datasets to investigate our hypothesis, as the TCGA datasets are large-scale RNA sequence datasets of breast cancer as well as other types of tumors. As for methods, the DESeq2 algorithm was used to compare differentially expressed genes between the two groups. When compared with other algorithms analyzing differentially expressed genes, DESeq2 shows the best performance in general [32]. To analyze the gene ontology, the DAVID database and GSEA were used. GSEA is still recommended to analyze gene ontology [33]. Based on these, our results are reliable and reproducible. Drug metabolizing enzymes such as ABCB5, CYP1A1 CYP4Z1, and CYP4A22 were significantly upregulated in the anthracycline-R samples in our analysis. Consistently, a study confirmed that enzymes including CYP1A1, CYP2C19, ABCB1, and other key transporters involved in drug resistance were significantly upregulated in the anthracycline-R breast cancer cell lines [34,35,36]. Interestingly, we found that anthracycline sensitivity was significantly associated with cysteine and methionine metabolism, while another study reported that wild-type MCF-7was significantly enrichedin the global metabolic processes such as cysteine and methionine metabolism, glutathione metabolism, and pyruvate metabolism when compared with the anthracycline-R MCF-7 [37].

Overall, our study highlightedtranscriptomic, genetic, and immune differences between the anthracycline-S and anthracycline-R samples. These results uncovered the potential molecular mechanisms underlying anthracycline resistance. Targeting these abnormalities might reverseanthracycline resistance and bring survival benefits for the anthracycline-R breast cancer patients.

## 5. Conclusions

In summary, through omics analysis of RNA sequencing data and genome sequencing data, we found transcriptomic difference between the anthracycline-S and anthracycline-R samples, with 204 upregulated genes and 1376 downregulated genes. Besides, we also found the difference in genetic alterations between the anthracycline-S and anthracycline-R patients. *TP53* mutation was observed in the anthracycline-S group while *CDH1* mutation was presented in the anthracycline-R group. We also detected that the immune status was different between the anthracycline-S and anthracycline-R groups. These results uncovered the potential molecular mechanisms underlying anthracycline resistance.

## Data Availability

Data are available on request from the authors.

## Supplementary Materials

The following supporting information can be downloaded at: https://www.mdpi.com/article/10.3390/biom12121834/s1, Supplementary Table S1. Chemotherapy regimens for breast cancer [38,39]. Supplementary Table S2. Differentially expressed genes between the anthracycline-S and anthracycline-R patients. Supplementary Table S3. Gene ontology analysis of differentially expressed genes. Supplementary Table S4. GESA on hallmarkers and KEGG pathways in the anthracycline-S and anthracycline-R patients.
