# Supplementary material for: Comprehensive Analysis of Transcriptomics and Genetic Alterations Identifies Potential Mechanisms Underlying Anthracycline Therapy Resistance in Breast Cancer
Source: Biomolecules. 2022 Dec 8;12(12):1834. doi: 10.3390/biom12121834 (PMC9775906; doi:10.3390/biom12121834)
Supplement: Supplementary file 1 [file biomolecules-12-01834-s001.zip › Supplementary Table S1. Chemotherapy regimens for breast cancer.pdf]

Supplementary Table S1. Chemotherapy regimens for breast cancer[38,39]

	Regimens for HER2-negative breast cancer	Regimens for HER2-positive breast cancer
**Preoperative/Adjuvant Therapy Regimens**	**TC**<br>(docetaxel plus cyclophosphamide)	**TH**<br>(paclitaxel and trastuzumab)
	**AC-T, dose dense**<br>(doxorubicin plus cyclophosphamide followed by paclitaxel)	**TCH**<br>(docetaxel, carboplatin, and trastuzumab)
	**AC**<br>(doxorubicin plus cyclophosphamide)	**TCHP**<br>(trastuzumab, pertuzumab, carboplatin, and docetaxel)
	**TAC**<br>(docetaxel, doxorubicin, and cyclophosphamide)	**AC-TH**<br>(doxorubicin plus cyclophosphamide followed by paclitaxel plus trastuzumab)
	**Oral CMF**<br>(oral cyclophosphamide plus methotrexate and fluorouracil)	**AC-THP**<br>(doxorubicin and cyclophosphamide followed by paclitaxel, trastuzumab, and pertuzumab)
	**IV CMF**<br>(IV cyclophosphamide plus methotrexate and fluorouracil)	**FEC-THP**<br>(FEC followed by docetaxel, pertuzumab, and trastuzumab)
	**FEC**<br>(fluorouracil, epirubicin, plus cyclophosphamide)	**THP-FEC-H**<br>(neoadjuvant docetaxel, trastuzumab, and pertuzumab followed by adjuvant FEC, followed by trastuzumab)
	**FEC** followed by weekly paclitaxel	**THP**<br>(docetaxel/paclitaxel, trastuzumab, and pertuzumab)
	**FEC** followed by weekly docetaxel	
	**TC**<br>(paclitaxel and carboplatin)	
	**EC**<br>(epirubicin and cyclophosphamide)	
	**Anthracyclines**<br>(doxorubicinand liposomal doxorubicin)	**THP**<br>(pertuzumab, trastuzumab and docetaxel)
**Recurrent/MetastaticTherapy Regimens**	**Taxanes**<br>(paclitaxel)	**THP**<br>(trastuzumab and Pertuzumab and paclitaxel/docetaxel)
	**Anti-metabolites**<br>(capecitabine and gemcitabine)	Ado-trastuzumab emtansine (T-DM1)
	**Microtubule inhibitors**<br>(vinorelbineand eribulin)	Fam-trastuzumab deruxtecan-nxki
	Sacituzumab govitecan-hziy	Tucatinib and trastuzumab and capecitabine
	Cyclophosphamide	Trastuzumab and docetaxel or vinorelbine

	Docetaxel	Trastuzumab and paclitaxel and or capecitabine
	Albumin-bound paclitaxel	Capecitabine and trastuzumab or lapatinib
	Epirubicin	Trastuzumab and lapatinib
	Ixabepilone	Neratinib and capecitabine
	**AC**<br>(doxorubicin and cyclophosphamide)	Margetuximab-cmkb and capecitabine or eribulin or gemcitabine or vinorelbine
	**EC**<br>(epirubicin and cyclophosphamide)	
	**CMF**<br>(cyclophosphamide, methotrexate and fluorouracil)	
	Docetaxel/capecitabine	
	**GT**<br>(gemcitabine and paclitaxel)	
	Gemcitabine/carboplatin	
	Carboplatin plus paclitaxel or albumin-bound paclitaxel	

References:

38. Gradishar, W. J.; M. S. Moran; J. Abraham;, R. Aft; D. Agnese; K. H. Allison; B. Anderson; H. J. Burstein; H. Chew and C. Dang. Breast cancer, version 3.2022, NCCN clinical practice guidelines in oncology. Journal of the National Comprehensive Cancer Network 20 **2022**, 691-722.

39. Gradishar, W. J. NCCN guidelines updates: Management of patients with her2-negative breast cancer. Journal of the National Comprehensive Cancer Network 20 **2022**, 561-65.
